# Management Strategies and Outcomes of Hemorrhagic Traumatic Brain Injury on Oral Anticoagulants

**DOI:** 10.7759/cureus.10508

**Published:** 2020-09-17

**Authors:** Evan M Krueger, Megan M Finneran, Michelle Smith

**Affiliations:** 1 Department of Neurosurgery, Carle Foundation Hospital, Normal, USA; 2 Department of Trauma, OSF Healthcare, Bloomington, USA

**Keywords:** traumatic brain injury, anticoagulation, antiplatelet, intracranial hemorrhage

## Abstract

Introduction: Traumatic brain injury (TBI) is common, and the frequency of patients taking oral anticoagulants is increasing. However the optimal initial triage, management, and long term care plans of hemorrhagic TBI patients taking oral anticoagulants is not clear.

Objectives: To determine the usage pattern of reversal agents for hemorrhagic TBI patients taking oral anticoagulants, and examine their characteristics and outcomes as compared to hemorrhagic TBI patients not taking these medications.

Methods: This was a single-center, retrospective, observational study. Included were adults with trauma categorization and traumatic intracranial hemorrhage (ICH) between April 1, 2017 and December 31, 2019. Patient age, type of ICH, initial Glasgow Coma Scale (GCS) score, oral anticoagulant prescribed pre-injury, anticoagulation reversal agent given, and hospital discharge disposition were recorded.

Results: For the entire sample size (n=111), the mean age and GCS were 71.6 years old and 13.8, respectively. Compared to patients not taking oral anticoagulants, patients taking oral anticoagulants were older (76.7 years old versus 69.1; *p*<0.01), had similar GCS scores (13.7 versus 13.9; *p*=0.69), had fewer subarachnoid hemorrhages (18.9% versus 37.8%; *p*=0.04), were less likely to discharge home (48.6% versus 73.0%; *p*=0.01), and had similar incidence of mortality (13.5% versus 6.7%; *p*=0.30). A total of 14/37 (37.8%) patients taking oral anticoagulants received reversal agents in the emergency department. Compared to patients taking oral anticoagulants and not given reversal agents, patients taking oral anticoagulants and given reversal agents had similar ages (78.8 years old versus 75.4; *p*=0.41), had similar GCS scores (12.9 versus 14.1; *p*=0.17), had similar ICH types (all *p*=1.0), were less likely to discharge home (48.6% versus 73.0%; *p*=0.01), and had higher incidence of mortality (28.6% versus 4.2%; *p*=0.05).

Conclusions: This limited data set did not show improved outcomes by giving reversal agents to hemorrhagic TBI patients taking oral anticoagulants. However, until more robust data is available, judicious use of reversal agents in this high-risk patient population should remain common practice.

## Introduction

The yearly incidence of traumatic brain injury (TBI) in the United States is 1.6 million, resulting in 290,000 hospitalizations [1]. In 2010, the yearly economic burden in the United States for non-fatal TBI was nearly $88 billion [2]. As the general population ages, more patients are being prescribed oral anticoagulants [3]. However, management strategies for anticoagulant induced side-effects, such as intracranial hemorrhage (ICH), are not fully validated. There is even further paucity of data on the appropriate triage, management, and long term care for hemorrhagic TBI patients taking oral anticoagulants.

The purpose of this study was to share a single-center experience with managing hemorrhagic TBI patients on oral anticoagulants. We sought to determine the usage pattern of reversal agents for hemorrhagic TBI patients taking oral anticoagulants, and examine their characteristics and outcomes as compared to hemorrhagic TBI patients not taking these medications. The aim of this study is to improve care and resource utilization in hemorrhagic TBI patients.

## Materials and methods

This was a single-center, retrospective, observational study of a prospectively maintained outcomes database that obtained IRB approval (#0000068). All consecutive patients from the healthcare system trauma registry at a Level II trauma center between April 1, 2017 and December 31, 2019 were queried. Inclusion criteria were: age ≥18, categorized trauma by the emergency department (ED), and the presence of any type of acute traumatic ICH. Exclusion criteria were: presence of pre-existing intracranial blood or mass lesion, and incomplete records. The study variables recorded were patient age, type of ICH, initial Glasgow Coma Scale (GCS) score, oral anticoagulant prescribed pre-injury, anticoagulation reversal agent given in the ED (including human blood products and synthetic agents), and patient hospital discharge disposition. Patient groups were categorized as taking oral anticoagulants (OAC), not taking oral anticoagulants (n-OAC), taking oral anticoagulants and given reversal agents (OAC-r), or taking oral anticoagulants and not given reversal agents (OAC-nr).

A sample of convenience was utilized. An *a priori* power analysis was not performed since consecutive patients were reviewed. To compare mean age and GCS, a student’s t-test was used. A Fischer’s exact test or chi-square test were used to compare type of ICH and hospital discharge disposition. All calculations were performed using SAS (version 9.4; SAS Institute Inc., Cary, NC, US). Means (range, ± 1 standard deviation) are reported. *P*≤0.05 was considered statistically significant.

## Results

A total of 111 patients met inclusion criteria. The mean age and GCS were 71.6 (23-95 years old, ±14.1) and 13.8 (3-15, ±2.5), respectively. There were 77/111 (69.4%) subdural hematomas (SDH), 35/111 (31.5%) traumatic subarachnoid hemorrhages (SAH), and 8/111 (13.8%) intraparenchymal hemorrhages (IPH). For hospital discharge disposition, 72/111 (64.9%) went home, 23/111 (20.1%) went to a skilled nursing facility (SNF), 4/111 (3.6%) went to in-patient rehab, and 2/111 (1.8%) were transferred to another facility directly from the ED. There were 10/111 (9.0%) mortalities, with a mean GCS score of 9.

Thirty-seven of 111 (33.3%) patients were taking oral anticoagulants prior to injury. The distribution of OAC were as follows: 17/37 (46.0%) clopidogrel, 9/37 (24.3%) warfarin, 8/37 (21.6%) apixiban, 2/37 (5.4%) dabigatran, 2/37 (5.4%) rivaroxaban, 1/37 (2.7%) aspirin-dipyridamole, and 1/37 (2.7%) ticagrelor. OAC were older than n-OAC (76.7 years old, 38-95, ±11.8; versus 69.1, 23-90, ±14.5; *p*<0.01). OAC had similar GCS scores compared to n-OAC (13.7, 6-15, ±2.6; versus 13.9, 3-15, ± 2.5; *p*=0.69). As compared to n-OAC, OAC had similar rates of SDH (27/37, 73.0%; versus 50/74, 67.6%; *p*=0.47), had fewer SAH (7/37, 18.9%; versus 28/74, 37.8%; *p*=0.04), and had similar rates of IPH (4/37, 10.8%; versus 4/74, 5.4%; *p*=0.44). As compared to n-OAC, OAC were less likely to discharge home (18/37, 48.6%; versus 54/74, 73.0%; *p*=0.01), were more likely to discharge to SNF or rehab (14/37, 37.8%; versus 13/74, 17.5%; *p*<0.01), and had similar incidence of mortality (5/37, 13.5%; versus 5/74, 6.7%; *p*=0.30).

A total of 14/37 (37.8%) patients taking oral anticoagulants received reversal agents in the ED (Table 1).

**Table 1:** Hemorrhagic Traumatic Brain Injury Patients on Oral Anticoagulation Medicine Given Reversal Agents. Abbreviations: IPH, intraparenchymal hemorrhage; FFP, fresh frozen plasma; GCS, glasgow coma scale; OAC, oral anticoagulant; PCC, prothrombin complex concentrate; pRBC, packed red blood cell; SAH, subarachnoid hemorrhage; SDH, subdural hematoma; SNF, skilled nursing facility; vit K, vitamin K

Age	Hemorrhage	GCS	OAC	Reversal Agent	Disposition
75	SAH	9	apixiban	PCC	mortality
80	SDH	6	apixiban	PCC	mortality
86	SDH	14	apixiban	PCC	SNF
61	IPH	15	clopidogrel	platelets	rehab
84	SDH	14	clopidogrel	platelets	SNF
86	SDH	15	clopidogrel	platelets	SNF
81	SDH	13	clopidogrel, warfarin	FFP, vit K	SNF
81	SAH	15	dabigatran	idarucizumab	home
88	SDH	14	rivaroxaban	platelets	SNF
85	SAH	15	warfarin	FFP	home
65	SDH	15	warfarin	vit K	home
74	SDH	15	warfarin	vit K, PCC	home
78	SDH	6	warfarin	FFP	mortality
79	SDH	15	warfarin	FFP, vit K, pRBC	mortality

Specifically, 6/9 (66.6%) patients taking vitamin-k antagonists, 4/10 (40%) patients taking factor X inhibitors, 4/19 (21.1%) patients taking antiplatelet medications, and 1/2 (50%) patients taking direct thrombin inhibitors pre-morbid were reversed. The OAC-nr group is summarized in Table 2.

**Table 2:** Hemorrhagic Traumatic Brain Injury Patients on Oral Anticoagulation Medicine not Given Reversal Agents. Abbreviations: IPH, intraparenchymal hemorrhage; GCS, glasgow coma scale; OAC, oral anticoagulant; SAH, subarachnoid hemorrhage; SDH, subdural hematoma; SNF, skilled nursing facility.

Age	Hemorrhage	GCS	OAC	Disposition
64	SDH	14	apixaban	home
63	SDH	15	apixaban	home
90	SDH	15	apixaban	home
87	SDH	15	apixaban	home
86	SDH	15	apixaban	SNF
69	SDH	15	aspirin, dipyridamole	home
38	IPH	15	clopidogrel	home
61	SAH	15	clopidogrel	home
84	SDH	14	clopidogrel	home
73	SDH	15	clopidogrel	home
83	SDH	15	clopidogrel	home
78	SDH	15	clopidogrel	mortality
72	IPH	12	clopidogrel	SNF
77	SDH	11	clopidogrel	SNF
76	SDH	12	clopidogrel	SNF
93	SDH	15	clopidogrel	SNF
86	SDH, SAH	15	clopidogrel	SNF
71	SDH	15	clopidogrel, rivaroxaban	home
88	SDH	15	clopidogrel, warfarin	home
95	SDH	15	dabigatran	home
78	SAH	15	ticagrelor	SNF
71	SAH	15	warfarin	home
52	IPH	7	warfarin	SNF

OAC-r had similar ages compared to OAC-nr (78.8 years old, 61-88, ±7.9; versus 75.4, 38-95, ±13.7; *p*=0.41). OAC-r had similar GCS scores compared to OAC-nr (12.9, 6-15, ±3.3; versus 14.1, 7-15, ±1.9; *p*=0.17). Compared to OAC-nr, OAC-r had similar rates of SDH (10/14, 71.4%; versus 17/23, 70.8%; *p*=1.0), SAH (3/14, 21.4%; versus 4/23, 16.7%; *p*=1.0 ), and IPH (1/14, 7.1%; versus 3/23, 12.5%; *p*=1.0 ). Compared to OAC-nr, OAC-r were less likely to discharge home (4/14, 28.6%; versus 14/23, 60.9%, *p*=0.05), had similar rates of discharge to SNF or rehab (6/14, 42.8%; versus 8/23, 34.8%; *p*=0.38), and had higher incidence of mortality (4/14, 28.6%; versus 1/23, 4.2%; *p*=0.05).

## Discussion

During initial triage of hemorrhagic TBI patients, early clinical decision making is often inferred from limited information. While outcomes of all TBI vary tremendously, mild TBI - defined as GCS 13-15 - have reported only 3.5% rates of neurosurgical intervention [4]. Furthermore, hemorrhagic mild TBI patients have reported only 1.5% rates of unexpected delayed neurosurgical intervention [5]. In our study, OAC and n-OAC patients surprisingly had similar GCS scores. Newer oral anticoagulants such as apixaban, dabigatran, or rivaroxaban have previously been reported to not have higher rates of traumatic ICH occurrence, progression, or death as compared to aspirin, clopidogrel, and warfarin [6]. Additionally, pre-injury antiplatelet usage has been shown to be associated with increased mortality [7]. Congruently, in our study, OAC patients were less likely to be discharged home as compared to n-OAC patients.

Whether to give reversal agents to hemorrhagic TBI patients is complex, and is based upon many factors including immediate and foreseeable risk of clinical and radiographic deterioration, and need for neurosurgical intervention. A clinician weighs these against reversal agent complications such as thromboembolism, financial cost, and potential limited availability and efficacy. In our study, there were no differences in age, GCS, and ICH types for OAC-r and OAC-nr. Despite their similarities, OAC-r had worse hospital discharge dispositions and higher incidence of mortality compared to OAC-nr. Our data however is limited, and any conclusions drawn exclusively from it should be viewed as equivocal. In a retrospective study, it was noted that while patients receiving platelet transfusions for pre-morbid aspirin or clopidogrel use had higher injury severity scores and lower GCS, their mortality was significantly higher as compared to patients not receiving platelet transfusions [7]. Platelet transfusion does not result in decreased expansion of non-operative traumatic SDH [8]. Conversely, others have reported improved outcomes and reduced mortality with platelet transfusions for hemorrhagic TBI patients on P2Y12 inhibitors [9]. For patients older than 60 years old with traumatic ICH, mortality is higher in patients taking vitamin-k antagonists compared to other types of anticoagulants, despite that they more commonly receive reversal agents [10]. As a potential alternative to traditional reversal agents, in an international multicenter trial of TBI patients with a GCS≤12 or any acute ICH, patients were randomized to receive one gram of tranexamic acid (TXA) load within three hours of injury followed by one gram TXA over eight hours versus placebo control. Those receiving TXA had a significant reduction in mortality compared to placebo (12.5% versus 14.0%, risk ratio 0.89), with a similar risk of vascular occlusive events [11]. Zero patients in our study received TXA. Ultimately despite the limited contrarian data, administration of reversal agents for hemorrhagic TBI patients should, at this time, remain common practice.

In most instances, immediately halting pre-injury oral anticoagulation after hemorrhagic TBI is indicated. However, the timing and method of reinitiating of oral anticoagulants remain unclear. Resuming antiplatelet therapy after traumatic ICH has reported re-hemorrhage risks of <1% if within 48 hours, and cumulative 4.7% if within two weeks [12]. Others have proposed reinitiating oral anticoagulants 9.5 days after injury as an appropriate balance of hemorrhagic and thromboembolic complications [13]. We did not explore timing of resumption of oral anticoagulants in our study.

Managing patients on oral anticoagulants with spontaneous ICH, by comparison, has more evidence available to support clinical decision making. The risk of recurrent spontaneous ICH appears to be centered on individualized risk factors including location of hemorrhage, patient’s age, cerebrovascular disease, need for continued anticoagulation, and genetics [14,15]. An international multicenter trial of non-surgical patients on antiplatelet therapy with acute spontaneous primary ICH, randomized patients to receive platelet transfusion plus standard medical therapy versus standard medical therapy alone. Patients receiving platelet transfusion had higher odds of death or dependence at three months (odds ratio 2.05, *p*=0.01) and a trend towards higher adverse events during their hospital stay [16]. Antiplatelet monotherapy can be reinitiated days after any spontaneous ICH, while the optimal resumption of other oral anticoagulants after non-lobar ICH has been suggested to range from 72 hours to four weeks [14,15]. Warfarin should not be continued for the treatment of nonvalvular atrial fibrillation after a warfarin-induced lobar ICH [14].

This study shared the experience of managing hemorrhagic TBI patients at a Level II trauma center. In the United States, there is a growing trend of regionalization of trauma hospitals despite that the majority of neurosurgeons do not practice at Level I trauma centers [17]. Most neurosurgeons may not have a robust blood bank in their armamentarium. Additionally, financial cost can unfortunately cloud clinical decision making. In 2020, the costs per patient to give prothrombin concentrate and andexanet-alpha were $5,670 and $22,129, respectively; the later exceeded Medicare total hospital reimbursement in 74% of patients by $7,604 [18]. There may be a select group of hemorrhagic TBI patients on oral anticoagulants whose risk of neurologic decline is less than the risk associated with administration of a reversal agent. Key areas of future focus should include improving resource utilization of scare, expensive, and potentially dangerous oral anticoagulant reversal agents.

There are several limitations to this study. Foremost, this was a retrospective observational study with a relatively small sample size, which carries inherent limitations. Next, our database did not record potential confounders including, (1) pupillary response, which may have resulted in very poor prognosis patients (e.g., GCS 3 or 4 with fixed unreactive pupils) being included for analysis, and (2) serum ethanol levels or other pre-existing medical comorbidities such as hematologic conditions or liver cirrhosis, that may have affected a patient’s coagulation profile and risk of hemorrhage, (3) the size of the traumatic ICH, and (4) the mechanism or severity of traumatic injury. Third, we lumped all oral anticoagulants and reversal agents together for statistical analysis, despite their significant heterogeneity. Another limitation is that we did not consider the clinicians’ justifications for giving a reversal agent, such as if it was based on clinical exam, laboratory values, or some combination. Fifth, we did not have a way to record where the patient was living prior to admission, and therefore a patient who was discharged to a skilled nursing facility may have actually returned to their pre-injury home. Lastly, our database did not record the patient’s clinical or functional status at discharge.

## Conclusions

In conclusion, we present a retrospective, single-center review of reversal strategies and short-term outcomes of TBI patients on oral anticoagulants. In a relatively small sample size, we showed OAC patients were older than n-OAC patients and tended to have worse hospital discharge dispositions. The decision to administer reversal agents did not appear to be dictated by age, GCS, or type of ICH. Receiving reversal agents was not associated with improved discharge disposition or lower mortality. However judicious use of reversal agents in this high-risk patient population should still remain common practice. For the foreseeable future, clinical decision making for hemorrhagic TBI patients on oral anticoagulants will be informed primarily from a collection of lower powered studies.
